# Promotion and induction of liver cancer by gut microbiome-mediated modulation of bile acids

**DOI:** 10.1371/journal.ppat.1007954

**Published:** 2019-09-05

**Authors:** Baolei Jia, Che Ok Jeon

**Affiliations:** 1 School of Bioengineering, Qilu University of Technology (Shandong Academy of Sciences), Jinan, China; 2 Department of Life Science, Chung-Ang University, Seoul, Republic of Korea; University of Utah, UNITED STATES

## Introduction

The human gastrointestinal tract is colonized by a complex and dynamic range of symbiotic microorganisms, collectively termed the gut microbiome, which defends the human host against pathogens and maintains metabolic homeostasis and immune balance [1]. However, although the gut microbiome directly benefits the host, it is also involved in the development of diseases [2], and there is mounting evidence of its contribution to both local and distant carcinogenesis in humans and animal models [3]. Local bacterial–induced carcinogenesis (i.e., gastrointestinal carcinogenesis) is exemplified by the induction of gastric cancer by *Helicobacter pylori* through its secretion of the virulence factor cytotoxin-associated gene A (CagA) [4]. Another example is that gut commensal *Escherichia coli* (*pks*^+^) and other *Proteobacteria* that produce colibactin promote colon cancer development [5]. In contrast, distal bacterial–induced carcinogenesis affects extraintestinal organs such as the liver, heart, adipose tissue, pancreas, lungs, brain, and cardiovascular system [6]. The liver, being closely connected to the gut via the hepatic portal vein, is the first recipient of gut microbiome metabolites and antigens, including bile acids (BAs), lipopolysaccharide (LPS), choline, indole derivatives, and short-chain fatty acids [7].

Changes in the gut microbiome contribute to the initiation and progression of liver diseases, including alcoholic fatty liver disease, nonalcoholic fatty liver disease (NAFLD), nonalcoholic steatohepatitis, cirrhosis, and liver cancer [8]. Among them, liver cancer is the most common primary hepatic malignancy and a leading cause of death in patients with cirrhosis [9]. Recent research has revealed the carcinogenic effects of small molecules produced by the gut microbiome that downregulate the immune system in the liver; these molecules include LPS, BAs, and lipoteichoic acid (LTA) [10–13]. LPS can activate Toll-like receptor (TLR) 4 to contribute to the pathogenesis of liver cancer [14]. BAs are common and important components of the gastrointestinal tract, where they emulsify lipids, cholesterol, and fat-soluble vitamins for absorption [15]. BAs were implicated as carcinogens as early as 1940 [16], when high levels of secondary BAs were shown to generate reactive oxygen and nitrogen species, disrupt cell membranes and mitochondria, and induce DNA damage and apoptosis—ultimately leading to colon cancer [17]. The relationships between gut microbiota, BAs, and liver diseases, including hepatic steatosis, NAFLD, nonalcoholic steatohepatitis, cirrhosis, and hepatocellular carcinoma (HCC), have been reviewed extensively [18, 19]. In the present opinion paper, we summarize recent studies describing the influence of the gut microbiome on liver cancer pathogenesis through BA-mediated regulation of the liver immune system. The findings summarized are expected to guide the development of strategies aimed at liver cancer prevention and clinical therapy by highlighting the gut microbiome as a therapeutic target.

### Microbial bile salt hydrolases initiate BA metabolism in the gut

BAs are classified as primary BAs (synthesized by the liver) and secondary BAs (products of the bacterial metabolism of primary BAs). Primary BAs undergo conjugation with taurine or glycine in the liver, remain in the biliary system, and are then secreted into the intestine, where they mediate lipid absorption [20]. Bacterial bile salt hydrolases (BSHs) catalyze the hydrolysis of conjugated BAs to produce free (deconjugated) BAs and amino acids in the gut [21]. BSH activity has been reported in a wide variety of bacterial species from gastrointestinal to nonintestinal origin [22]. The enzymes can be expressed in the cytoplasm of bacteria or in the extracellular region [23]. Currently, >30 BSHs have been characterized biochemically, most of which preferentially hydrolyze glyco-conjugated BAs. Few enzymes preferentially hydrolyze tauroconjugates or both glyco- and tauro-conjugated BAs [24]. As a key mediator of BA transformations, BSHs have been considered a promising target for the rational manipulation of the gut microbiota to benefit host physiology [21].

### *Clostridium* species in the gut microbiome produce secondary BAs by 7α-dehydroxylation

In the gut, bacteria from *Clostridium* cluster XIVa (including *Clostridium scindens*, *C*. *hiranonis*, and *C*. *hylemonae*) and *Clostridium* cluster XI (*C*. *sordellii*) can convert the primary 7α-hydroxyl BAs, cholic acid (CA), and chenodeoxycholic acid (CDCA), into the secondary BAs deoxycholic acid (DCA) and lithocholic acid (LCA), respectively, through a 7α/β-dehydroxylation reaction catalyzed by enzymes encoded by the BA-inducible (*bai*) operon [25]. CA is imported into the bacterial cell via BaiG (a proton-dependent transporter) and ligated to coenzyme A (CoA) by the ATP-dependent CoA-ligase BaiB or ATP-independent CoA-transferases BaiF and BaiK [26]. Subsequently, 7α-hydroxysteroid dehydrogenases convert host primary BAs to 7-oxo-BAs. The resulting BA-CoA conjugates are then converted to a C3-oxo BA intermediate through oxidation of their C3-hydroxyl group by the NAD(H)-dependent 3α-hydroxysteroid dehydrogenase BaiA [27]. Two other enzymes, BaiCD and BaiH, catalyze the formation of a C–4 = C–5 bond in 7α-hydroxyl BAs (CA and CDCA) and 7β-hydroxyl BAs (ursodeoxycholic acid and 3α,7α-dihydro-5β-cholan-24-oic acid), respectively. BaiE (a 7α-dehydratase) catalyzes the 7α-dehydration reaction, the rate-limiting and irreversible step in this pathway. Finally, BaiN reduces the intermediates to form DCA or LCA, which are secreted from the bacterial cell and therefore released into the host intestine [28].

### Secondary BAs promote obesity-associated liver cancer through PGE_2_-mediated suppression of antitumor immunity

**Fig 1:**
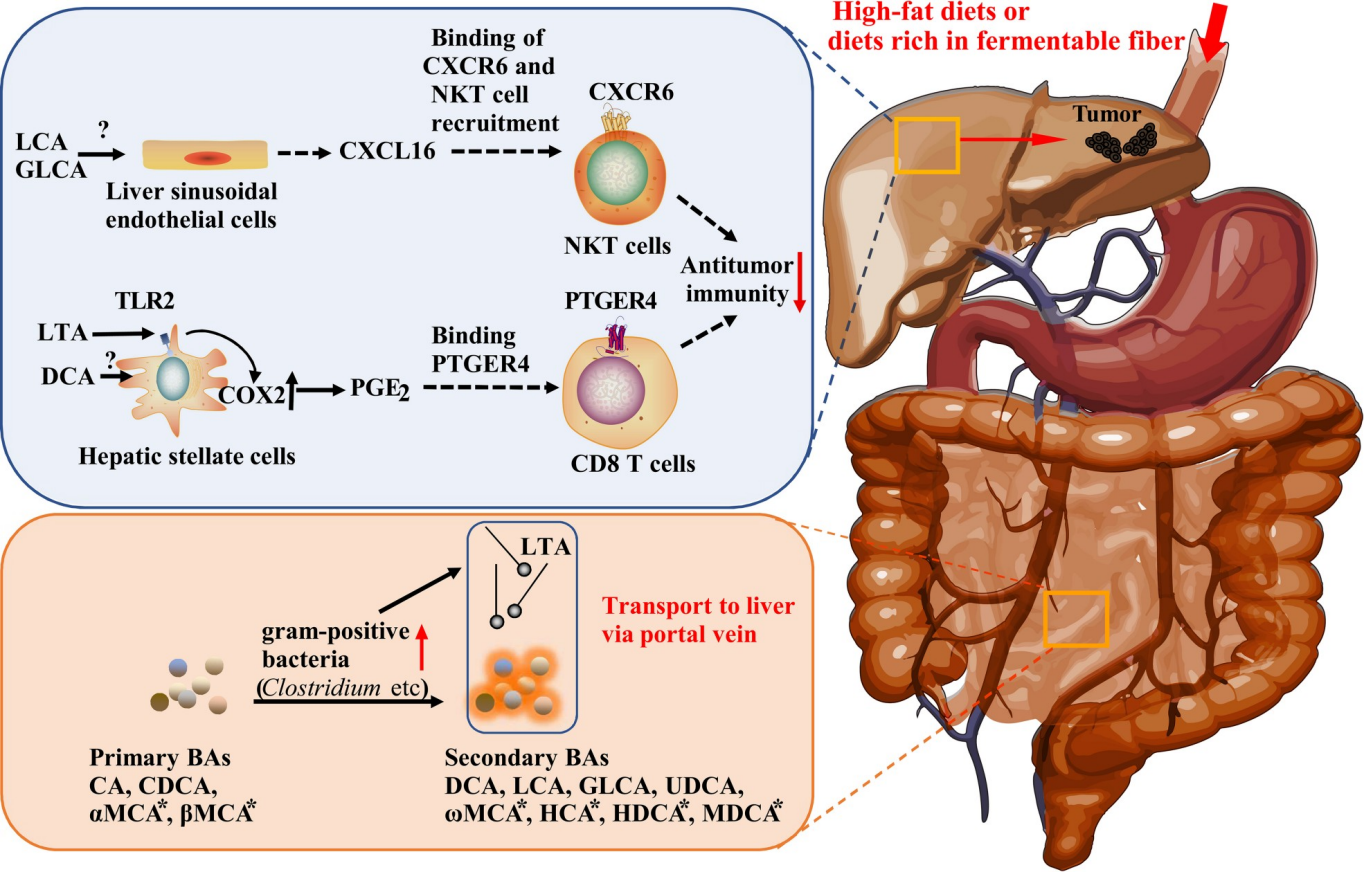
Schematic representation of the two pathways through which the gut microbiome putatively promotes liver cancer via antitumor immunosuppression. BAs marked with an asterisk are from rats and mice only. BA, bile acid; CA, cholic acid; CDCA, chenodeoxycholic acid; COX2, cyclooxygenase 2; CXCL16, chemokine (C-X-C motif) ligand 16; CXCR6, C-X-C chemokine receptor type 6; DCA, deoxycholic acid; GLCA, glucuronic acid; HCA, hyocholic acid; HDCA, hyodeoxycholic acid; LCA, lithocholic acid; LTA, lipoteichoic acid; MCA, muricholic acid; MDCA, murideoxycholic acid; PGE_2_, prostaglandin E_2_; PTGER4, prostaglandin E receptor 4; UDCA, ursodeoxycholic acid.

Diet-induced obesity induces the proliferation of gram-positive gut microbiota. In a study of mice fed a high-fat diet (HFD), a single species similar to the DCA-producing strain *C*. *sordellii* from *Clostridium* cluster XI constituted greater than 12% of the fecal bacteria, whereas another bacterial taxon resembling *Clostridium* cluster XIVa (*C*. *hylemonae* and *C*. *scindens*) comprised 0.5% of the fecal bacteria. The increase in the abundance of these strains corresponded to an increase in DCA levels (Fig 1) [11, 13]. Further, LTA, a major constituent of the cell wall of gram-positive bacteria, has also been shown to accumulate in the livers of HFD-fed mice in the presence of DMBA (7,12- dimethylbenz(a)anthracene, a chemical carcinogen) that can give rise to hepatocellular carcinoma (HCC) [11]. Both DCA and LTA cooperatively induce the senescence-associated secretory phenotype (SASP) of hepatic stellate cells to produce various inflammatory and protumorigenic factors, including interleukin-6, growth-regulated oncogene-alpha, chemokine (C-X-C motif) ligand (CXCL) 9, and prostaglandin E_2_ (PGE_2_), leading to a tumorigenic microenvironment that promotes liver cancer development in mice. Among these SASP-produced factors, PGE_2_ is critical in tumor development [11]. PGE_2_ production is regulated through the detection of LTA by the innate immunity ligand TLR2. LTA and DCA accumulation cooperatively enhance the TLR2-mediated signals to induce the overexpression of cyclooxygenase 2 (COX2) (but not that of COX1), which catalyzes the rate-limiting step in PGE_2_ biosynthesis. COX2 and the prostaglandin cascade are associated with inflammatory diseases and carcinogenesis through their suppression of dendritic cells, natural killer T (NKT) cells, and type-1 immunity, thereby promoting tumor immune evasion [29]. In the liver, COX2-mediated PGE_2_ production suppresses antitumor immunity through the prostaglandin E receptor 4 (PTGER4) on CD8 T cells, thereby promoting liver cancer progression [11]. Indeed, gut sterilization by antibiotics significantly suppresses the SASP of hepatic stellate cells and attenuates its liver cancer–promoting effects [11, 13]. Together, these results highlight the significant contribution of the gut microbiome in promoting obesity-associated liver cancer.

### Secondary BAs regulate liver cancer via NKT cells

Ma and colleagues demonstrated that primary and secondary BAs additionally mediate the opposing effects on liver tumor growth through NKT cells (Fig 1) [10]. In mice, the primary BAs, CDCA and taurocholic acid, stimulate the expression of CXCL16 (chemokine [C-X-C motif] ligand 16) in liver sinusoidal endothelial cells via an unknown mechanism [10]. C-X-C chemokine receptor type 6 (CXCR6), which is the receptor for CXCL16, modulates the survival and accumulation of NKT cells in the liver [30]. CXCR6 accumulation in liver NKT cells increases interferon-gamma production, contributing to tumor growth inhibition. In contrast, secondary BAs, such as LCA or omega-muricholic acid (ω-MCA), inactivate liver sinusoidal endothelial cells and reduce the accumulation of NKT cells. Indeed, the inhibition of *Clostridium* species by vancomycin enhances liver NKT cell accumulation, whereas colonization by *C*. *scindens* rapidly decreases the amount of liver NKT cells, further indicating that *Clostridium* species are responsible for enzymatically converting primary BAs to secondary BAs [30]. These findings implicate the interplay of BAs, CXCL16, CXCR6, and NKT cells in the regulation of liver cancer pathogenesis in mice. In humans, *CXCL16* expression is positively correlated with the levels of CDCA levels but inversely correlated with those of glycolithocholate (a secondary BA), suggesting that liver cancer is similarly regulated in humans.

### Soluble fiber–induced liver cancer is microbiome-dependent and the oncogenic microbiome is transmissible

A recent study showed that feeding TLR5-deficient mice (*T5*KO mice) a diet enriched with soluble fiber, such as inulin, pectin, and fructooligosaccharides, resulted in the induction of cholestatic liver cancer [12]. The mice with liver cancer demonstrated pre-existing microbiota dysbiosis, indicating that the induced liver cancer was microbiome-dependent. The accumulation of fiber-fermenting bacteria and proteobacteria was also observed in the gut of *T5*KO mice. The fiber-fermenting bacteria included members of Clostridia, especially the *Clostridium* cluster XIVa, which are capable of enzymatically converting primary BAs to secondary BAs [31]. Furthermore, cohousing of the dysbiotic mice with wild-type (WT) mice led to the development of liver cancer in both groups, while the two groups were maintained on an inulin-containing diet. The cause of liver cancer in cross-fostered WT mice supported the function of gut microbiota in soluble fiber–induced liver cancer. Taken together, these data highlight the role of the gut microbiota in fiber-induced liver cancer in mice and further demonstrate that the liver cancer–inducing bacteria are transmissible.

## Discussion

Recent studies have demonstrated direct associations between the gut microbiome, secondary BAs, immune system, and liver cancer [10, 11, 13]. Two pathways through which the gut microbiome regulates liver cancer via secondary BAs have been described, as shown in Fig 1. To elucidate the pathway of secondary BA-mediated tumor progression through suppression of antitumor immunity, the obesity-associated HCC model was used [11]. The population of *Clostridium* cluster XI was strikingly increased in the mice model [13]. Further, Ma and colleagues used WT mice induced by tumor cell injection or spontaneously HCC-induced MYC transgenic mice to study the regulation of live cancer by BAs via NKT cells [10]. The study showed that *C*. *scindens* colonization in the gut reduced the amount of hepatic NKT cells and promoted liver tumor growth in the mice injected with tumor cells [10]. Furthermore, Singh and colleagues recently demonstrated that clostridia, including *Clostridium* cluster XIVa, were enriched in the gut of *T5*KO mice with pre-existing microbiota dysbiosis fed fermentable fiber-enriched compositionally defined diets [12]. These studies imply that the liver cancer–promoting effect of clostridia is conditioned and may be related to the specific mouse model or presence of gut dysbiosis. Further, cohousing of the dysbiotic *T5*KO mice with WT mice demonstrated that the oncogenic bacteria could be transmitted to susceptible mice [12]. On the basis of these findings, we propose that *Clostridium* species harboring the *bai* operon and other related strains should be considered oncogenic microbiota capable of promoting and/or inducing liver cancer.

The studies reviewed herein constitute an initial exploration of the connection between the gut microbiome and liver cancer. The diet appears to have an important influence on the gut microbial composition. Significantly increased amounts of *Clostridium* cluster XI were found in the fecal bacterial community of mice fed HFD compared with that in mice fed a normal diet. Further, all HFD-fed mice subject to oncogenic stimuli developed liver cancer, whereas only 5% of normal diet–fed mice developed tumors of the lung but not of the liver [13]. This result was confirmed by Yamada and colleagues, who showed that C57BL/6J mice fed with a steatohepatitis-inducing high-fat diet demonstrated increased abundance of *Clostridium* cluster XVIII; C57BL/6J mice fed the high-fat diet developed nonalcoholic steatohepatitis that further progressed into liver cancer without the administration of chemical carcinogens [32]. In addition to HFD, dietary fiber was also found to strongly influence the gut bacterial composition. Refined soluble fibers induced liver cancer in *T5*KO mice with microbiota dysbiosis. However, dietary intervention with insoluble fiber (cellulose) has been shown to protect *T5*KO mice from soluble fiber–induced liver cancer. Furthermore, feeding of HFD together with inulin induced both dysbiosis and liver cancer in WT mice [12]. The results were further confirmed by Janssen and colleagues [33], who showed that when a mouse model of NAFLD was fed fermentable dietary fiber and guar gum, enhanced hepatic inflammation and fibrosis were induced via alteration of the gut bacteria. In sum, these studies suggest that improper diet, especially HFD, is a strong factor that synergizes with *Clostridium* species to promote liver cancer in mice.

Among the approximately 50 secondary BAs identified in human feces to date, DCA and LCA are the most abundant [34]. The role of various secondary BAs in promoting liver cancer is not clear. Armstrong and Cameron reported in 1991 that hepatic nodules and liver cancer was induced in rats fed DCA [35]. The study additionally demonstrated that DCA was the key secondary BA promoting liver cancer development [11, 13]. Recently, Ma and colleagues reported that ω-MCA plays a significant role in promoting liver cancer [10] However, ω-MCA is present in the gut of rats and mice but not in that of humans [20]. ω-MCA is transformed from β-MCA in a cooperative manner by three strains: a *Eubacterium lentum* strain and two atypical *Fusobacterium* sp. strains [36] In contrast, DCA is catalyzed from CA by gut *Clostridium* clusters XI and XVIa. The findings of Ma and colleagues additionally suggest that tauro-ω-MCA, taurodeoxycholic acid (TDCA), taurolithocholic acid (TLCA), and tauroursodeoxycholic acid (TUDCA) are involved in promoting liver cancer. Among them, TDCA, TLCA, and TUDCA can be found in the gut of both mice and humans. In this respect, the significance of the various BAs in promoting liver cancer requires further clarification.

Various secondary BAs can activate nuclear receptors (including farnesoid X receptor [FXR], pregnane X receptor, and the vitamin D receptor) and G protein-coupled receptor (TGR5) to initiate a series of signaling events and regulate hepatic metabolism or disease. Among these receptors, FXR and TGR5 are highly expressed in cells of the innate immune system [37]. However, the mechanism through which the secondary BAs initialize immunosuppression is still unclear. DCA and LTA cooperatively induce the SASP in hepatic stellate cells. LTA can bind TLR2, a receptor that plays an important role in the innate immune system. The role of DCA in initiating the production of inflammatory and protumorigenic factors by hepatic stellate cells requires elucidation. Furthermore, the mechanisms by which the primary and secondary BAs regulate CXCL16 expression remain unclear. CXCL16 can be expressed as a chemokine in soluble form or a membrane protein, because the protein is composed of a domain with a small and extracellular cytokine, a membrane-spanning domain with a single α-helix, and a domain with a cytoplasmic extension [38]. Further studies should be carried out to clarify the mechanism underlying BA-mediated regulation of CXCL16 expression.

### Future perspectives

Current research has shed light on the effects of the gut microbiome and diet on liver health in humans, with important clinical and nutritional implications. The data from these studies demonstrate that clostridial colonization caused by HFDs or other diets may promote and induce tumor growth and that this effect may also be transmissible. As the gut microbiota is one of the drivers of carcinogenesis in patients with liver cancer, clostridia harboring the *bai* operon can be considered a promising target for liver cancer prevention. The relative abundance of these bacteria in the gut can be quantified by real-time PCR [39]. Antibiotics represent one of the promising strategies for the prevention of liver cancer because they target several bacteria and pathways in the gut–liver axis [19]. Vancomycin treatment, which depletes *Clostridium*, can increase liver NKT cell accumulation and block liver cancer development [10, 13]. However, vancomycin is seldom used in patients because of the risk of several potentially severe adverse effects. Both rifaximin and norfloxacin can also be potentially used for humans in the treatment of liver cancer [19]; however, the risk of antibiotic resistance and implication for patient safety limit their clinical application. Some nonantibiotic small molecules may be more promising; for example, difructose anhydride III (DFA III) treatment can decrease both deconjugation and 7α-dehydroxylation activity, which may substantially reduce HCC development in mice [13]. β-acids from hops (*Humulus lupulus*) have also been shown to attenuate HCC in inulin-fed mice by inhibiting bacterial fermentation without impacting gut bacterial loads [12]. These molecules specifically target 7α-dehydroxylation or related processes in bacterial fermentation, representing a potentially safe and effective clinical approach to liver cancer prevention.

The current studies also suggest that liver health is influenced by the activity of the gut microbiome intervention because fiber-induced liver cancer was shown to be transmissible to WT mice via cohousing or cross-fostering [12]. There are currently no data indicating that fecal microbiota transplantation (FMT) is effective in the prevention of liver cancer. However, FMT has been shown to alleviate steatohepatitis induced by HFD in mice [40], implying that FMT may also be an effective therapeutic option for liver cancer prevention. Probiotics that reshape the gut microbial community may suppress tumor growth in mice by inducing the production of anti-inflammatory metabolites from beneficial bacteria [41]. We proposed that certain probiotics in a competitive relationship with 7α-dehydroxylating bacteria may also exert a beneficial effect in preventing HCC growth: 7α-dehydroxylating bacteria promote liver cancer by suppression of antitumor immunity, whereas *Bifidobacterium* present in the gut has been reported to facilitate antitumor immunity [42]. Further, *Bifidobacterium* supplements in the diet suppress chemically induced liver tumors [43], suggesting that this bacterium is potentially useful as a probiotic for preventing liver tumor growth.

Furthermore, the composition of the gut microbiota is closely related to the antitumor efficacy of immune checkpoint inhibitors. Several commensal bacteria, such as *Akkermansia muciniphila*, *Bifidobacterium* species, *Bacteroides* species, and *Enterococcus hirae*, promote the therapeutic efficacy of anti–PD-L1 (programmed cell death 1 ligand 1); in contrast, some bacteria, such as *Roseburia intestinalis* and *E*. *coli*, are negatively correlated with the efficacy of anti-PD-1 and anti-CTLA-4 therapy [44]. It is therefore accepted that the gut microbiota influences hepatic innate immunity via the gut–liver axis; accordingly, gut microbiota is considered to potentially affect the efficacy of immunotherapies against liver cancer. Further studies should aim to identify bacteria that influence the efficacy of immunotherapies against liver cancer, with a specific focus on the putative involvement of 7α-dehydroxylating bacteria in this process.
